# Supplementary material for: Geobacter sulfurreducens Extracellular Multiheme Cytochrome PgcA Facilitates Respiration to Fe(III) Oxides But Not Electrodes
Source: Front Microbiol. 2017 Dec 12;8:2481. doi: 10.3389/fmicb.2017.02481 (PMC5732950; doi:10.3389/fmicb.2017.02481)
Supplement: Supplementary file 1 [file Data_Sheet_1.PDF]

Supplemental Figures

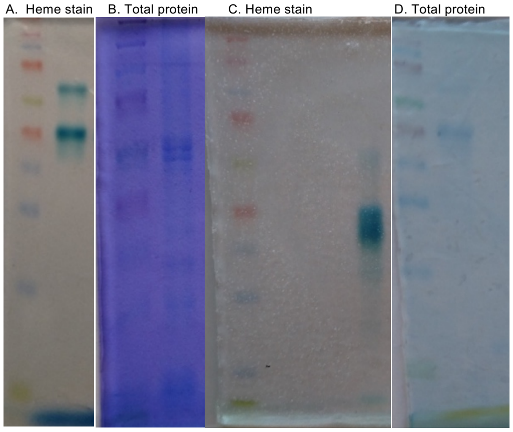

S1. Protein fractions obtained from nickel-NTA affinity columns and separated by SDS-PAGE. (A) purified PgcA stained by peroxidase activity ("heme stain"), and same samples stained for total protein (B). The two forms were pooled for most experiments. (C) Heme stain of sample post gel filtration chromatography to obtain small form. (D) Total protein stain post gel filtration chromatography. Protein ladder is Spectra broad range protein ladder 26623 from ThermoFisher Scientific. Note that due to different running times and shrinkage of gels in different stains, ladders are not aligned along the four images.

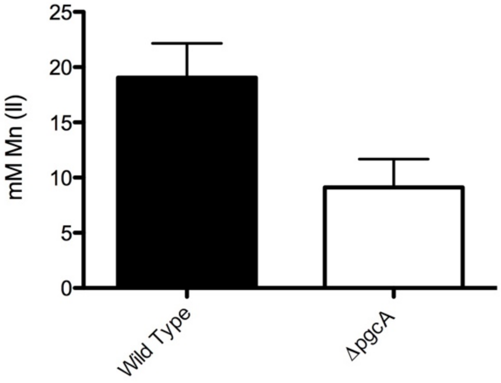

S2. Manganese reduction by wild type *G. sulfurreducens* and Δ*pgcA* mutant. A student's unpaired t-test supports a significant difference between the amount of manganese reduced at 72 hours post inoculation.

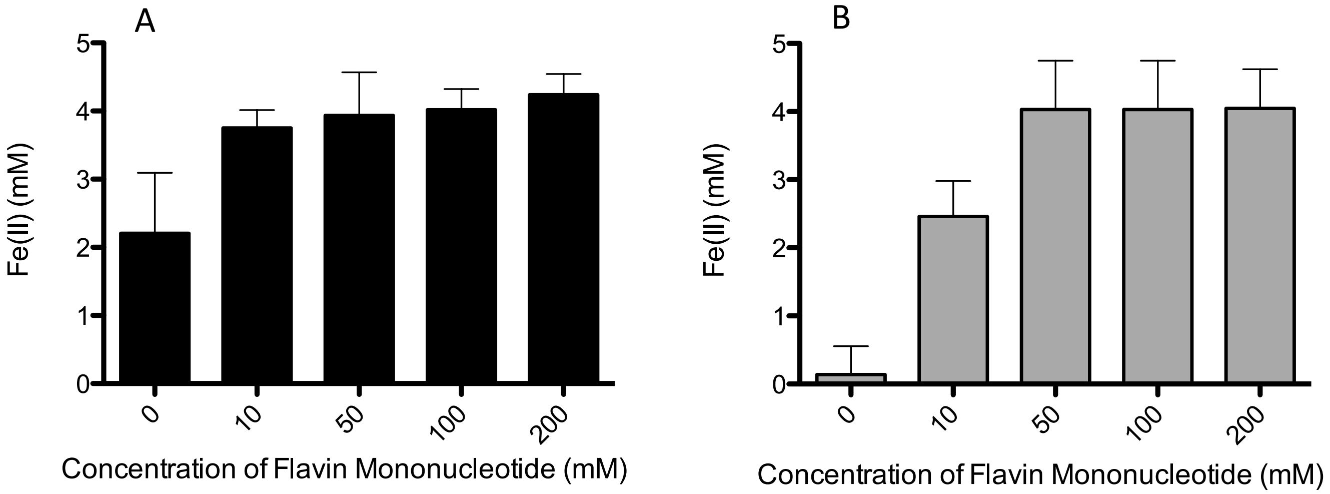

S3. Effects of increased FMN during Fe(III) oxide reduction assay. A. Wild type *G. sulfurreducens* and B. Δ*pgcA*. In both cases, addition of levels over 50 μM did not accelerate reduction rates further.

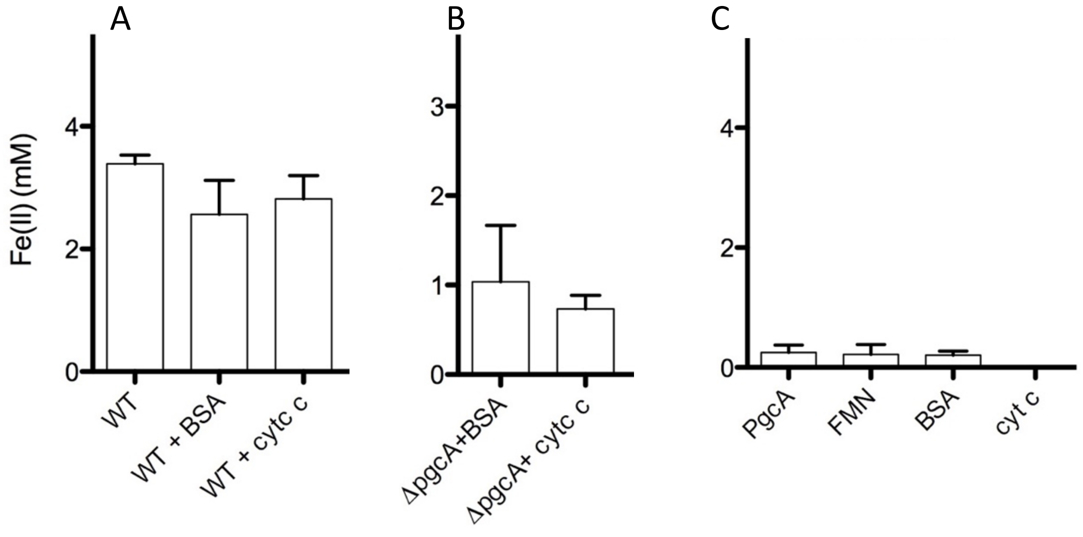

S4. Controls for stimulation of Fe(III) oxide reduction by added proteins and compounds. (A). Wild-type *G. sulfurreducens* with other proteins or shuttles added; BSA, Bovine Serum Albumin, and cyt *c*, horse heart cytochrome c, each added at 12 μM concentrations. Wild type with no additives is included for comparison. (B) Same controls, with Δ*pgcA* cells. (C) No-cell controls showing lack of reduction in the absence of cells. Like PgcA and FMN, horse heart cytochrome *c* were added to the assay in oxidized form.
